# Determining expression changes of ANO7 and SLC38A4 membrane transporters in colorectal cancer

**DOI:** 10.1016/j.heliyon.2024.e34464

**Published:** 2024-07-11

**Authors:** Elaheh Mohandesi Khosroshahi, Mazaher Maghsoudloo, Hossein Fahimi, Khatere Mokhtari, Maliheh Entezari, Maryam Peymani, Mehrdad Hashemi, Runlan Wan

**Affiliations:** a Department of Genetics, Faculty of Advanced Science and Technology, Tehran Medical Sciences, Islamic Azad University, Tehran, Iran; b Farhikhtegan Medical Convergence Sciences Research Center, Farhikhtegan Hospital Tehran Medical sciences, Islamic Azad University, Tehran, Iran; c Key Laboratory of Epigenetics and Oncology, The Research Center for Preclinical Medicine, Southwest Medical University, Luzhou 646000, Sichuan, China; d Department of Cell and Molecular Biology & Microbiology, Faculty of Biological Science and Technology, University of Isfahan, Isfahan, Iran; e Department of Biology, Faculty of Basic Sciences, Shahrekord Branch, Islamic Azad University, Shahrekord, Iran; f Department of Oncology, The Affiliated Hospital, Southwest Medical University, Luzhou 646000, China; g Key Laboratory of Medical Electrophysiology, Ministry of Education & Medical Electrophysiological Key Laboratory of Sichuan Province, (Collaborative Innovation Center for Prevention of Cardiovascular Diseases), Institute of Cardiovascular Research, Southwest Medical University, Luzhou 646000, China

**Keywords:** Colorectal cancer, Membrane transporters, ANO7, SLC38A4, Bioinformatic analysis, RT-qPCR, Cancer biomarkers

## Abstract

Membrane transporters are proteins responsible for facilitating the movement of molecules within biological membranes. They play a vital role in maintaining cellular homeostasis by regulating the transport of nutrients, ions, and other molecules into and out of cells. Our aim is to identify biomarkers in colorectal cancer using membrane transporter proteins. We utilized COAD TCGA data for this purpose. Subsequently, we conducted differential gene analysis and feature selection using membrane transporter proteins. Furthermore, we identified two potential genes, including ANO7 and SLC38A4. To validate the expression profiles of ANO7 and SLC38A4, key genes in this context, RT-qPCR was employed on colorectal cancer samples and adjacent normal tissues. Additionally, utilizing GEPIA2, Kaplan-Meier survival analysis, and cBioPortal, we assessed the status of these genes in various cancers, examining their methylation and mutation patterns. In conclusion, we suggest that ANO7 and SLC38A4 serve as prognostic biomarkers in colorectal cancer.

## Highlights

• Expression changes in ANO7 and SLC38A4 membrane transporters in CRC highlight their potential role in disease progression.
• Identification of candidate genes in CRC by integrating bioinformatics and clinical oncology approaches.
• Down-regulation of ANO7 and SLC38A4 genes in CRC using RT-qPCR validation.
• Evaluation of survival analysis, methylation, and mutation patterns of ANO7 and SLC38A4 across various cancers.
• An interdisciplinary approach transforms cancer understanding, promising advances in diagnosis, treatment, and prognosis.

## 1 Introduction

Colorectal cancer (CRC) is a type of cancer that begins in the colon or rectum, which are parts of the digestive system. It usually starts as small, benign clumps of cells called polyps, which can become cancerous over time [1]. Membrane transporters are proteins responsible for facilitating the movement of molecules within biological membranes. They play a vital role in maintaining cellular homeostasis by regulating the transport of nutrients, ions, and other molecules into and out of cells. Membrane transporters are classified into several families based on their structure and function, including ATP-binding cassette (ABC) transporters, solute carrier (SLC) transporters, and ion channels [2].

Recent studies have highlighted the involvement of membrane transporters in cancer and chemoresistance [[3], [4], [5], [6], [7], [8]]. Membrane transporters and channels (collectively the transportome) govern cellular influx and efflux of ions, nutrients, and drugs [9,10]. Cancer cells, in their pursuit for rapid growth and proliferation, exhibit increased demand for nutrients such as amino acids, glucose, and lipids. Membrane transporters play a crucial role in regulating the uptake of these nutrients and are often dysregulated in cancer cells [5]. ABC transporters are a group of membrane transporters that utilize ATP hydrolysis to transport a wide range of substrates across biological membranes [[11], [12], [13]]. SLC transporters, on the other hand, facilitate the transport of various substrates across biological membranes using 12 transmembrane domains, playing a role in the transport of amino acids, sugars, and other molecules [2]. Disruption in the regulation of membrane transporters in cancer cells presents an opportunity for developing novel therapeutic strategies. These transporters can act as importers, mediating the entry of drugs into cells, or as exporters, pumping drugs out of cells, leading to chemoresistance [3]. Therefore, targeting membrane transporters involved in drug uptake or efflux could enhance the efficacy of chemotherapy and overcome chemoresistance.

Several membrane transporters have been identified as potential therapeutic targets for treating cancer. For instance, studies have shown that Na+/K + ATPase plays a role in cisplatin uptake in ovarian cancer cells [3]. Inhibiting Na+/K + ATPase can reduce cisplatin uptake and increase chemoresistance [3]. Similarly, V-ATPase has been implicated in cisplatin uptake in epidermoid cancer cells [3,14]. Inhibiting V-ATPase can reduce cisplatin uptake and increase chemoresistance. Several membrane transporters have also been identified as potential therapeutic targets for treating CRC. For example, Na+/K + ATPase has been shown to be involved in cisplatin uptake in CRC cells [15]. Inhibiting Na+/K + ATPase can decrease cisplatin uptake and increase chemoresistance [15]. Similarly, V-ATPase has been implicated in cisplatin uptake in CRC cells. Inhibiting V-ATPase can reduce cisplatin uptake and increase chemoresistance [15].

With mounting evidence demonstrating the significant role of interactions between genes and proteins in understanding the molecular mechanisms of cancer, introducing a new concept of clinical systems in cancer research becomes crucial. Integrating systems biology, clinical sciences, omics-based technologies, bioinformatics, and computational sciences is essential to enhance the diagnosis, treatments, and prediction of diseases. Bioinformatics in cancer is a vital component of clinical systems in cancer and serves as a primary tool and approach for conducting systematic cancer research in clinical medicine [16].

Anoctamin 7 (ANO7) is a member of the anoctamin family, which comprises Ca2+-activated Cl– channels. This family consists of ten isoforms [17]. Some members of the anoctamin family, such as ANO1, have been implicated in the development of CRC. ANO1 has been identified as a target of honokiol, which inhibits the proliferation of CRC cells [18]. Similarly, Li et al. found that ANO9 downregulation is critical in the tumorigenesis and progression of CRC [19]. ANO7 has been observed to be downregulated in metastatic disease, and decreased protein expression has been associated with high-grade prostate cancer [20,21].

SLC38A4 is a member of the solute carrier proteins (SLC) superfamily and functions as a system amino acid transporter [22,23]. It is also known as neutral amino acid transporter 4 (SNAT4). Amino acids are essential for the survival and growth of rapidly proliferating cells, including embryonic and cancer cells [24]. Consequently, amino acid transporters are typically expressed at high levels in these proliferative cells [25]. This gene is downregulated in colorectal cancer and may have a tumor-suppressive role [26].

Our aim in this study is to initially identify membrane transporters using TCGA data for CRC. Additionally, we aim to identify the pathways associated with the candidate genes through co-expression network analysis. Finally, the expression levels of two candidate genes (ANO7 and SLC38A4) will be validated using RT-qPCR in CRC samples compared to adjacent normal tissues.

## 2 Methods and materials

### 2.1 Population and Sample studied

A total of 25 fresh tissue samples from patients diagnosed with CRC, along with 25 adjacent normal tissue samples, were collected with the confirmation of a specialist physician (Table 1). These samples were obtained following a thorough examination and criteria reported from affected individuals after obtaining informed consent. The control subjects in this study were the same individuals from the patient group who underwent tissue sampling from the tumor's margin. Subsequently, the fresh tissues were transferred to the designated laboratory under liquid nitrogen conditions. Sampling was conducted at Imam Khomeini Hospital in Tehran. (Samples were purchased from the Cancer Institute of Imam Khomeini Hospital, and all ethical considerations were managed by Imam Khomeini Hospital. Furthermore, since the samples were purchased, no collaboration was added to the protocol. Additionally, the relevant letter from the hospital and patients' consent approval were obtained from Imam Khomeini Hospital [27]). For the inclusion and exclusion criteria, examples were related to cancer patients who did not receive any form of treatment or chemotherapy. This study was reviewed and approved by the School of Pharmacy and Pharmaceutical Sciences, Islamic Azad University, Tehran Medical Sciences with ethics ID IR.IAU.PS.REC.1402.138. for the sake of completeness, the raw clinical data has been included in Table S1.

**Table 1:** The demographic and baseline clinic-philological characteristic of samples.

Variable		Frequency (Percentage%)
Gender	Male	12 (48 %)
	Female	13 (52 %)
Age (years)	≥50	18 (72 %)
	<50	7 (28 %)
Stage	I	3 (12 %)
	II	9 (36 %)
	III	8 (32 %)
	IV	5 (20 %)

### 2.2 TCGA data analysis

For investigating gene expression levels and variations, RNAseq data related to CRC from The Cancer Genome Atlas (TCGA) with project ID TCGA-COAD was employed. Initial data download and primary preprocessing were conducted using the R programming language along with packages such as TCGAbiolinks, edgeR, and limma. The CRC samples from TCGA encompassed 41 normal samples and 480 cancerous samples. Following data download, genes with an expression count per million (CPM) less than 10 in 70 % of the samples were removed, considering genes with near-zero or zero expression across samples. CPM stands for count per million, which divides the count of each gene in a sample by the total counts associated with that sample, normalized to a million. If a gene has near-zero or zero expression, its resulting CPM will be less than 10. Subsequently, post-filtering genes with near-zero or zero expression, normalization of data was performed using the Trimmed Mean of M-values (TMM) method. Previous studies have demonstrated that TMM normalization is a robust and reliable method for normalizing library size, effectively addressing composition biases, and preserving biological variability when assessing gene expression differences in RNAseq data [28]. Additionally, the data was transformed to a logarithmic scale base 2, utilizing the normalized expression matrix from the previous steps. The differential expression of all genes between normal and cancerous samples was then computed using a linear model approach implemented in the Limma R package (version: 3.54.2), with significance criteria set at FDR <0.01 and |LogFC| > 1. Initially, clinical data associated with samples, determining the status of whether a sample is cancerous or normal, was extracted. After identifying the status of each sample, using the limma package, the differential expression between cancerous and normal samples for all genes, including those associated with membrane transporters, was calculated. Furthermore, we conducted searches using keywords 'membrane transporter proteins' and 'protein classes' to identify the most suitable resource for this study. Subsequently, we discovered that the Human Protein Atlas online database (https://www.proteinatlas.org/) categorizes all proteins. Additionally, we downloaded the list of membrane transporter proteins from the human protein atlas (https://www.proteinatlas.org/humanproteome/proteinclasses).

### 2.3 Feature selection

Feature selection (FS) is fundamental in the development of machine learning models, as it plays an important role in improving performance and avoiding overfitting by carefully selecting a subset of features, either variable or predictor. The main goal of FS is to identify the most informative and influential features to achieve optimal results. This method is used in various fields, including biology and medicine, where it is used for tasks such as gene selection, biomarker identification, and drug exploration [29]. In our study, we used FeatureSelect software (FSS) [30] to identify the most distinct genes between normal and tumor samples in the COAD dataset. FSS is a specialized tool for feature or gene selection that uses filter, wrapper, and ensemble methods. FSS has 11 common methods, including Ant Colony Optimization (ACO), Cuckoo (CUK), GA (Genetic algorithm), Imperialist Competitive Algorithm (ICA), Learning Automata (LA), Heat Transfer Optimization (HTS), World Competitive Contest (WCC), Particle Swarm Optimization (PSO), Forest Optimization Algorithm (FOA), Discrete Symbiotic Optimization Search (DSOS), and League Championship Algorithm (LCA), that allow the generation of various statistical measures. Using FSS, we identified a small subset of genes in the COAD dataset that effectively discriminated between normal and tumor samples. It is noteworthy that all parameters in FSS were maintained at their default settings. Additionally, we utilized DisGeNET (https://www.disgenet.org/) [31] to identify the list of reported genes associated with COAD.

### 2.4 Analysis of survival (expression correlation with mortality rate)

To investigate the correlation between ANO7 and SLC38A4 expression and the mortality rates of CRC patients, clinical data from the TCGA dataset were utilized. Initially, the expression levels of ANO7 and SLC38A4 in cancer samples were categorized into two groups, High and Low, based on their mean expression in cancer samples. Subsequently, clinical data was integrated with these expression categories. Finally, utilizing the Cox regression analysis, the relationship between the expression levels of ANO7 and SLC38A4 and the survival outcomes of the patients was assessed. This analysis aimed to elucidate whether the expression levels of these genes correlate with the survival rates of individuals under study in the context of CRC.

### 2.5 Co-expression network

To identify pathways associated with ANO7 and SLC38A4, co-expression networks were constructed. Initially, the Pearson correlation coefficient was computed between the expression levels of ANO7, SLC38A4, and all expressed genes in CRC using TCGA data. Genes exhibiting a strong correlation with ANO7 and SLC38A4 (R > 0.5 and P < 0.01) were selected for inclusion in the co-expression network. Subsequently, these selected genes were incorporated into the co-expression network to elucidate pathways associated with the genes present in this network. MsigDB (https://www.gsea-msigdb.org/gsea/msigdb) and Enrichr (https://maayanlab.cloud/Enrichr/) online tools were employed to identify these pathways based on gene sets within the co-expression network. Furthermore, the expression levels of ANO7 and SLC38A4 were investigated in cancer samples compared to adjacent normal tumor tissue to ascertain differential expression patterns. This comparative analysis aimed to discern the expression alterations of ANO7 and SLC38A4 in CRC tissues in contrast to adjacent normal tissues.

### 2.6 Evaluating ANO7 and SLC38A4 expression in Diverse cancer types

Our study aimed to assess the expression levels of ANO7 and SLC38A4 across various cancer tissues, employing robust tools like UALCAN web resource (https://ualcan.path.uab.edu/). We meticulously analyzed RNA-seq data from TCGA and compared them with normal tissues sourced from the GTEx database.

### 2.7 Evaluation of ANO7 and SLC38A4 expression level in COAD and READ

The assessment of ANO7 and SLC38A4 expression in COAD and READ was conducted using the UALCAN web resource for CRC OMICS data analysis. ANO7 and SLC38A4 genes were inputted, and the extracted results encompassed sample samples, cancer stages, gender, and histological subtypes in CRC.

### 2.8 Mutational landscape analysis

Using cBioPortal (http://www.cbioportal.org/) and TCGA Pan-Cancer Atlas Studies, we scrutinized mutations in ANO7 and SLC38A4. We examined mutation frequencies, types, and Copy Number Alterations (CNAs) in these genes, pinpointing their genomic locations and delving into the realm of point mutations.

### 2.9 DNA methylation analysis

Exploring epigenetic nuances, we studied DNA methylation patterns using data from SMART App (http://www.bioinfo-zs.com/smartapp). Focusing on COAD cancer from TCGA, we deciphered the relationship between ANO7/SLC38A4 expression and methylation patterns.

### 2.10 Confirmation through Real-Time quantitative PCR analysis in CRC

To confirm the expression of ANO7 and SLC38A4 in CRC, several steps were taken. Initially, both tumor and normal tissues from CRC patients were utilized to extract total RNA, employing the RNX-Plus kit from CinnaGen in Iran. Subsequently, this RNA was reversed into complementary DNA (cDNA) using the RevertAid first-strand cDNA synthesis kit from Thermo Fisher Scientific in the USA. Following this, quantitative reverse transcription PCR (RT-qPCR) was conducted in duplicate for each sample using the Real-Time PCR Detection System LightCycler 96 by Roche in the USA, adhering to the manufacturer's guidelines. The focus of the analysis centered on the genes of interest, ANO7 and SLC38A4, while GAPDH was used as the reference gene. Primer sequences utilized for each gene can be found in Table S2.

### 2.11 Statistical analysis

For data from Real-Time PCR, REST software was utilized, complemented by Independent-sample *t*-Test, ANOVA, ROC curve analyses, and 2^-ΔΔCt^ method for statistical significance. Survival-related assessments employed the log-rank test, while Prism - GraphPad software generated comparative group graphs.

## 3 Results

### 3.1 The significant downregulation of numerous membrane transporters in CRC has been observed

**Fig. 1:**
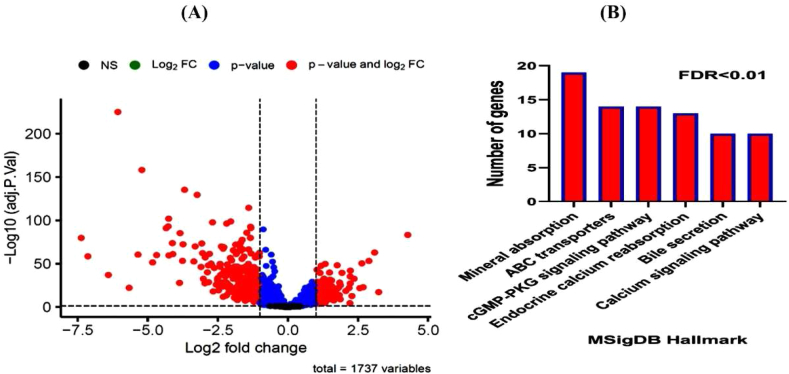
(A) Genes Exhibiting Significant Differential Expression based on logFC>1 and FDR<0.01 Criteria. (B) The identified pathways associated with cellular transport and translocation.

In the initial stage, the TCGA data underwent normalization, and the expression levels of membrane transporters in cancerous samples were investigated in comparison to normal samples. Our preliminary findings demonstrated that among 2138 genes associated with membrane transporters (Table S3), 1737 genes exhibited expression within the TCGA dataset for CRC samples (Table S4). Differential expression analyses revealed that 101 genes (Table S5) linked to membrane transporters exhibited significant and substantial upregulation (logFC>1 and FDR<0.01) in cancerous samples compared to normal ones (Fig. 1A). Conversely, 298 genes (Table S6) associated with membrane transporters exhibited decreased expression (logFC < −1 and FDR<0.01). The results suggest that the identified genes associated with membrane transporters could potentially play a role in colorectal malignancy. Subsequently, 298 downregulated genes were scrutinized, and their associated pathways were delineated. Pathway analysis revealed that a majority of the downregulated genes are involved in pathways such as Mineral absorption, ABC transporters, cGMP-PKG signaling, Endocrine, calcium, Bile secretion, and Calcium signaling. Each of these pathways (Mineral absorption [32], ABC transporters [33], cGMP-PKG signaling [34], and Calcium signaling [35]) is somehow involved in colorectal cancer. As illustrated in Fig. 1B, most of the identified pathways are linked to cellular transport and translocation mechanisms.

### 3.2 Feature selection

**Fig. 2:**
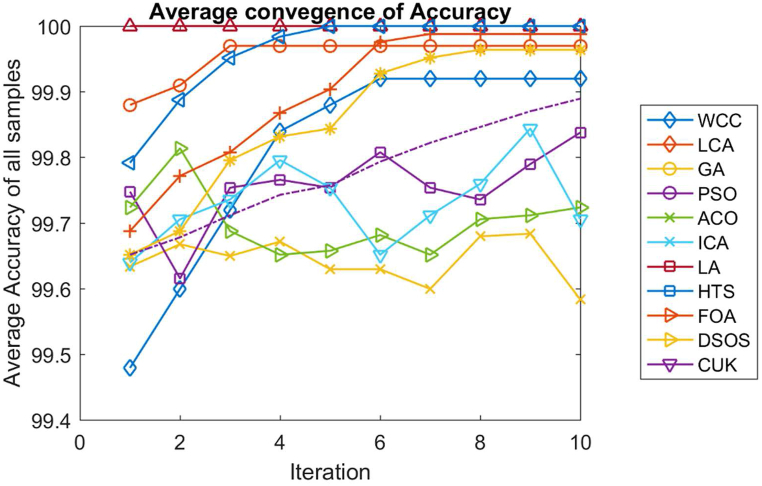
The plot of average accuracy for FSS. It illustrates the average convergence of accuracy for all algorithms within FSS.

We used the DEG expression matrix as the input of the feature FSS and set the maximum feature parameter to 10. In 10.13039/100031017FSS, we chose Support Vector Machine (SVM) as the classifier type. To ensure robust model training and evaluation, we split the dataset such that 80 % of the data was allocated for training purposes, while the remaining 20 % was considered for testing the model's performance. Fig. 2 graphically shows the average accuracy convergence across all methods employed in FSS. This visualization is to show the trends of effectiveness and convergence of different feature selection approaches in increasing classification accuracy.

The maximum accuracy for all methods was one hundred percent (100 %). Five methods, including ACO, GA, ICA, LA, and PSO, selected 10 genes each. Four methods, including CUK, HTS, LCA, and WCC, selected 8 genes each. The FOA method selected 6 genes. Interestingly, the DSOS method selected only four genes, which was the lowest number among all methods. Table 2 displays a list of genes selected using FSS. Subsequently, we extracted the reported genes associated with COAD from the DisGeNET database and identified new genes within the sets obtained from various FSS methods. The list of reported and novel genes associated with COAD is presented in Table S7. We observed that WCC and DSOS had the maximum reported genes in COAD. In DSOS, four genes were selected using FSS, while in WCC, eight genes were selected. Therefore, we considered the gene list from the WCC method. Among the eight genes selected in WCC, six were reported in COAD, and the remaining two, SLC38A4 and ANO7, were novel.

**Table 2:** The list of selected genes using FSS methods.

FSS method	List of selected genes
WCC	ABCA9-STX1A-KCND3-SLC38A4-IFITM2-SLC11A2-NR1H4-ANO7
LCA	SLC35D1-SLC6A8-CLIC5-KPNA2-RIC3-CLCN4-MEP1B-SLC44A4
GA	JPH2-TTYH3-REG3A-SLC17A9-ADRB2-SLC25A23-SLCO4C1-SLC15A1-SCNN1G-CLCA1
PSO	SLCO1B3-SLC12A8-NLGN4X-HBB-SLC22A11-JPH1-NPSR1-TRPA1-SCAMP2-SLC17A9
ACO	SHISA9-CNNM2-SIDT1-CNTN2-PIRT-HBA2-ANK2-SCNN1G-SLC8A1-KCNG1
LA	SLC35G1-NLGN1-SLC26A3-CLCN2-ATG9B-SLC9A3-FLNB-SYP-STRA6-MAGI2
HTS	GPER1-SORCS1-TRPV3-TMEM59-XKRX-SLC9A3-MS4A1-ADGRB3
DSOS	SLC7A5-SLC6A20-SLCO4A1-STRA6
CUK	SLC30A4-CDC20-PIRT-ARRB1-TRPM4-SLC28A2-SLC23A1-ADGRL2
ICA	SLC9A7-SUN2-MEP1B-CLDN23-GJB3-REG3A-CYGB-KCNMB1-SLC15A1-FXYD6
FOA	SLCO4C1-CLRN3-NGB-TMEM59-ADRB2-TSPAN18

### 3.3 The correlation between the expression of ANO7 and SLC38A4 genes and the mortality rate of patients

**Fig. 3:**
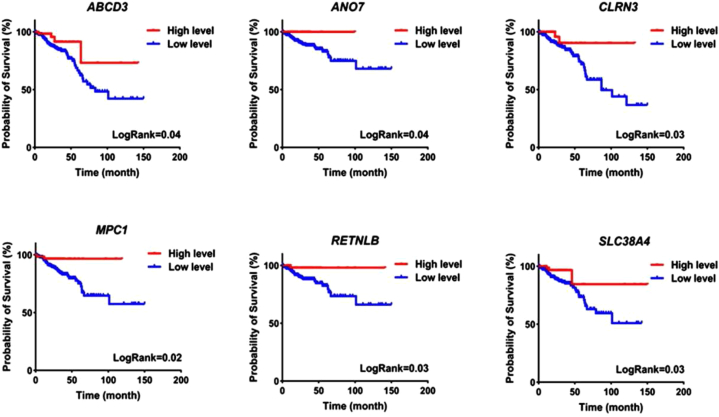
K-M plot illustrating the impact of each gene on CRC.

We explored the association between the expression of the identified 298 downregulated genes and the patients' mortality rates using clinical data from TCGA and Cox regression analysis. The results indicated that six downregulated genes associated with membrane transporters—MPC1, ABCD3, SLC38A4, CLRN3, RETNLB, and ANO7—were notably associated with favorable patient prognosis (Table 3). These findings suggest that downregulated genes could serve as prognostic biomarkers. To validate these results, the association between the expression of MPC1, ABCD3, SLC38A4, CLRN3, RETNLB, and ANO7 with patients' mortality rates was assessed through Kaplan-Meier curves. The outcomes demonstrated a significant correlation between increased expression of MPC1, ABCD3, SLC38A4, CLRN3, RETNLB, and ANO7 genes and reduced mortality rates among patients (Fig. 3). These results indicate that the expression levels of these genes, associated with membrane transporters, can serve as predictive biomarkers. To advance our research, in the previous step (feature selection section), we identified two genes, SLC38A4 and ANO7, which emerged as potential biomarkers in CRC. Furthermore, these genes exhibited favorable HRupper criteria in survival analysis. Consequently, we have decided to utilize these genes for future analyses.

**Table 3:** Cohort of 6 membrane transporter-related genes associated with favorable patient prognosis.

Name	HRupper	HRlower	HR	LogRank	Beta	Term
MPC1	0.797744	0.408537	0.570884	0.00103	−0.56057	ENSG00000060762
ABCD3	0.88197	0.50868	0.669806	0.004113	−0.40077	ENSG00000117528
SLC38A4	0.95706	0.448956	0.655498	0.001657	−0.42236	ENSG00000139209
CLRN3	0. 81688	0.482534	0.627832	0.000753	−0.46548	ENSG00000180745
RETNLB	0.884521	0.392311	0.589073	0.009659	−0.52921	ENSG00000163515
ANO7	0.929536	0.414432	0.620668	0.002074	−0.47696	ENSG00000146205

### 3.4 Identification of pathways associated with SLC38A4 and ANO7

**Fig. 4:**
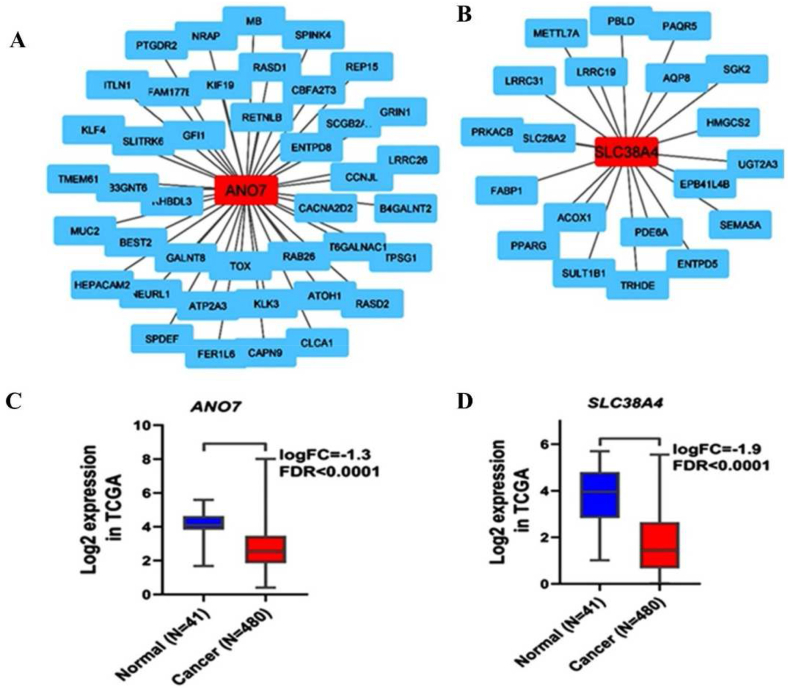
(A) The gene expression network of ANO7 and (B) SLC38A4 with their associated genes. (C–D) Differential gene expression between the tumor tissue and healthy tissue.

The co-expression network revealed a significant correlation of 20 genes, each with a correlation coefficient above 0.5, collectively associated with the expression level of SLC38A4 (Fig. 4B). Enrichment analysis for the 20 genes linked to SLC38A4 indicated their involvement in pathways such as PPAR signaling and Bile secretion. Conversely, 41 genes exhibited co-expression with ANO7 (Fig. 4A), but enrichment analysis did not identify significant pathways associated with this gene set. Additionally, bioinformatics data demonstrated a significant reduction in the expression levels of both SLC38A4 and ANO7 in cancer samples compared to normal, as depicted graphically in Fig. 4C and D.

### 3.5 Expression analysis of ANO7 and SLC38A4 in pan-cancer

**Fig. 5:**
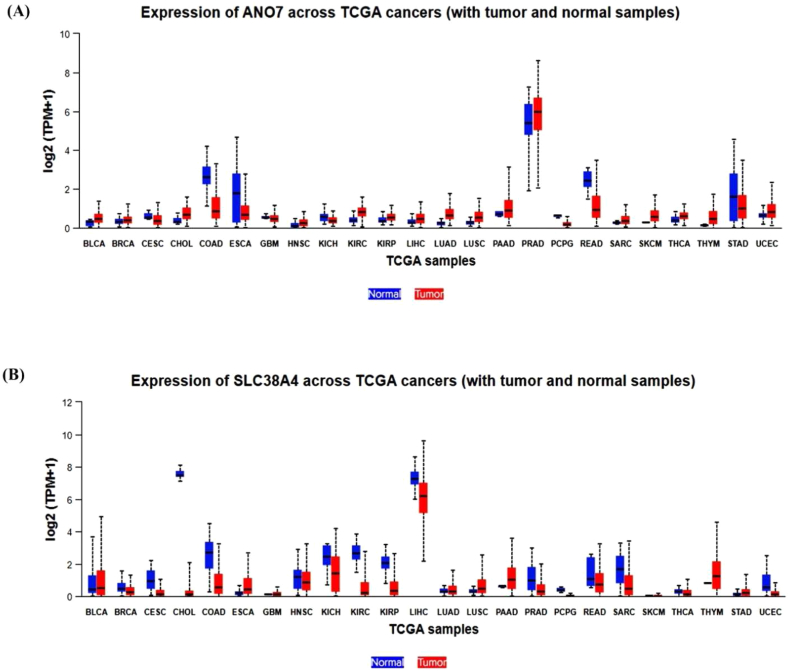
The levels of *ANO7 and SLC38A4* expression display variation among different types of cancer. (A) Depicts the profiles of *ANO7* levels in tumors compared to those in normal tissues. (B) Depicts the profiles of *SLC38A4* levels in tumors compared to those in normal tissues.

We conducted an analysis of *ANO7 and SLC38A4* expression in cancer and normal tissues using data from TCGA and GTEx datasets. Notably, our findings indicated a significant downregulation of *ANO7 and SLC38A4* expression in cancer tissues compared to normal tissues across various cancer types, including, but not limited to, Rectum adenocarcinoma (READ), Stomach Adenocarcinoma (STAD), Head and neck squamous cell carcinomas (HNSC), and colorectal cancer (COAD), as depicted in Fig. 5.

### 3.6 Evaluation of ANO7 and SLC38A4 gene expression level in COAD and READ

**Fig. 6:**
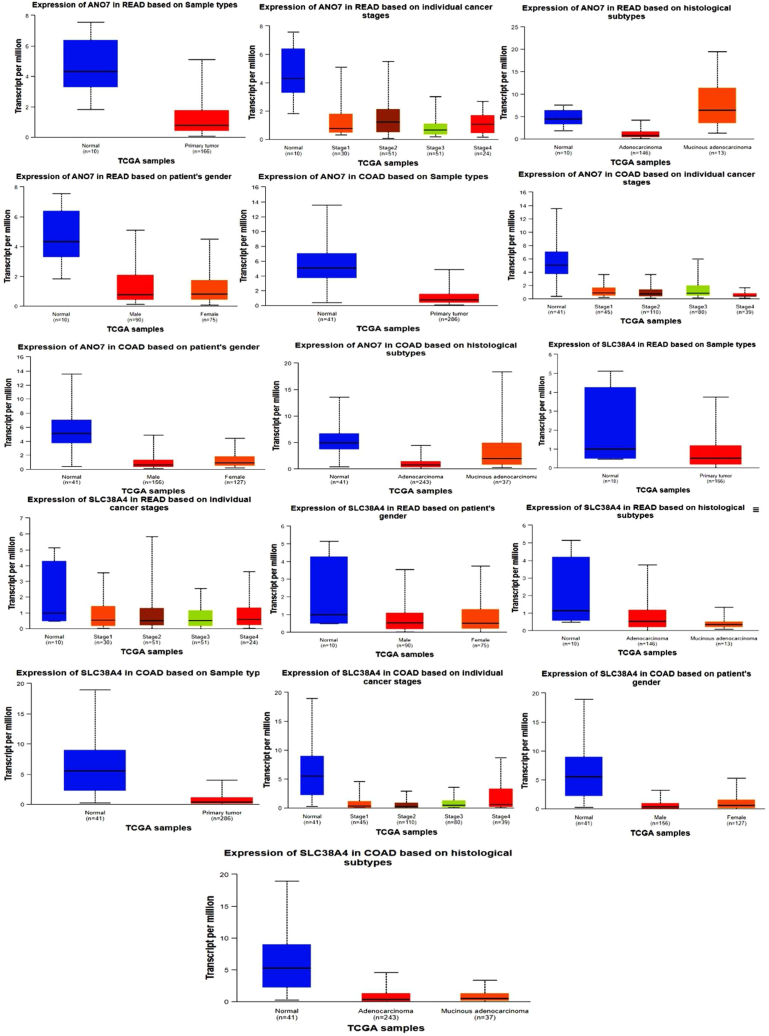
*ANO7* and *SLC38A4* expression based on TCGA-READ and TCGA- COAD data based on individual cancer stages, gender in patient, histological subtypes and sample type (p-value <0.05).

We used UALCAN web resource to analyze CRC OMICS data. We entered the *ANO7 and SLC38A4* gene as input and extracted the results of sample samples, cancer stages, gender and histological subtypes in CRC. The results of this analysis are shown in Fig. 6. All plots in Fig. 4 show that the expression level of *ANO7 and SLC38A4* gene is down-regulated. Hence, this gene can be introduced as a diagnostic candidate biomarker in CRC because its expression is down-regulated in all stages and subtypes of CRC as well as in different genders (Fig. 6).

#### 3.6.1 Gene alteration of ANO7 and SLC38A4 in pan-cancer

**Fig. 7:**
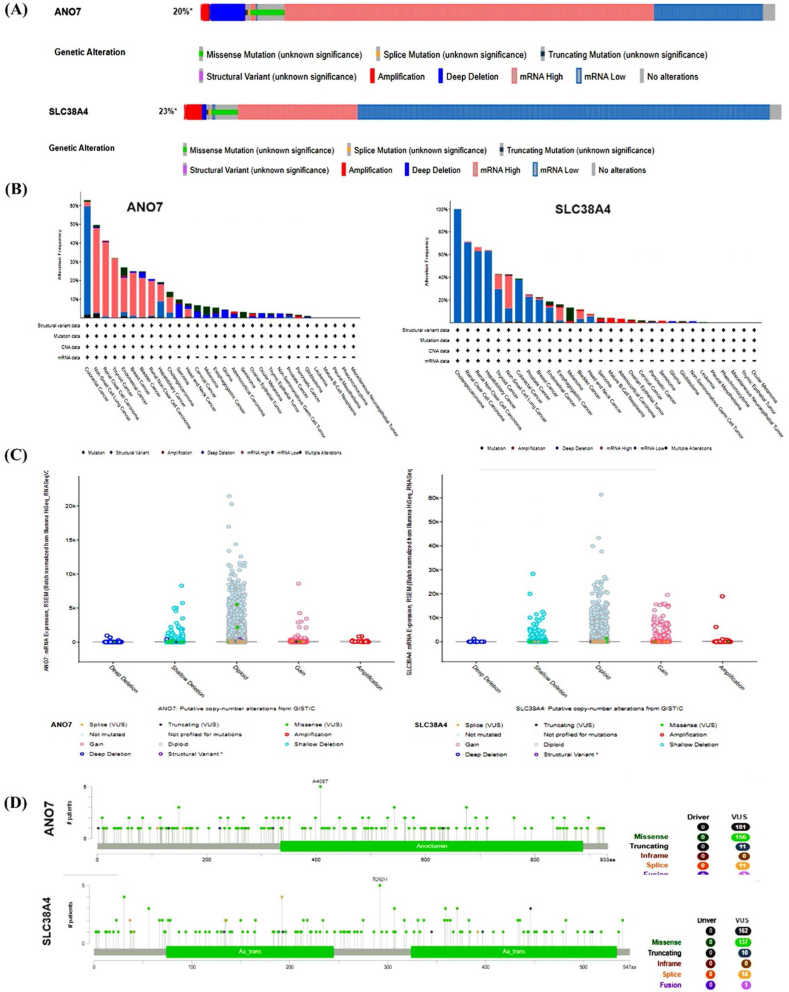
*ANO7 and SLC38A4* mutation landscape. (A) The connection between various cancer types and the expression of ANO7 and SLC38A4, highlighting those mutations predominantly correlated with RNA expression. (B) The potential changes in copy numbers of ANO7 and SLC38A4 as determined by GISTIC in numerous TCGA cancer types, accessed through the cBioPortal database. (C) Mutational profile of *SERPINE1* across different cancer types. (D) Point Mutation in ANO7 and SLC38A4.

The complex interplay between gene mutations, CNAs, and the progression of tumors is widely recognized. In our exploration of *ANO7 and SLC38A4* gene modifications using the cBioPortal platform, a discernible pattern has come to light. For *ANO7* Within this framework, the predominant type of alteration is denoted as “mRNA high," followed by “mRNA low " modifications and for *SLC38A4* Within this framework, the predominant type of alteration is denoted as " mRNA low ", followed by " mRNA high" modifications (Fig. 7A–D). These discoveries enhance our comprehension of the genetic foundations that drive tumor advancement and open doors for additional research and therapeutic investigations. Additionally, mutations have been scrutinized in a point-wise manner.

### 3.7 ANO7 and SLC38A4 showed significant downregulation in CRC tissues

**Fig. 8:**
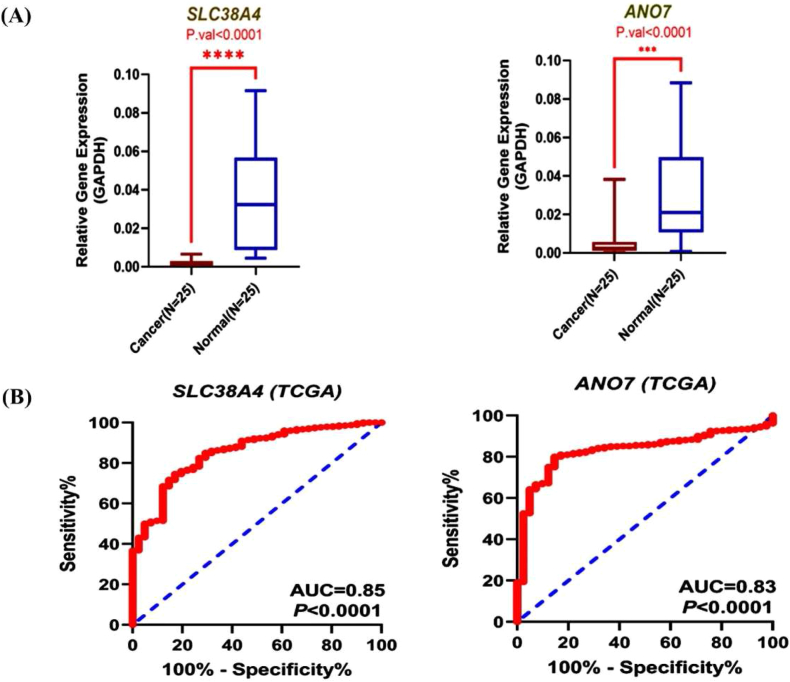
Expression levels of ANO7 and SLC38A4 biomarkers in clinical samples were analyzed using qPCR. The results of the qPCR analysis for ANO7 and SLC38A4 were presented in (A). Receiver operating characteristic (ROC) curves were generated for ANO7 and SLC38A4, and the results were presented in (B) (AUC: area under the curve).

In the context of the escalating significance of ANO7 and SLC38A4 as promising and innovative prognostic, immunomodulatory, and therapeutic biomarkers in CRC, we conducted a comprehensive analysis of their expression levels in CRC tissues (n = 25) alongside adjacent normal tissues (n = 25) using RT-qPCR. Our investigation revealed a significant reduction in the expression levels of both ANO7 and SLC38A4 in CRC tissues compared to adjacent normal tissues, as prominently illustrated in Fig. 8 (p = 1e−04).

## 4 Discussion

The relationship between CRC and membrane transporters is a topic of significant interest in oncology and cellular biology. CRC is a complex disease involving various genetic, environmental, and cellular factors [1]. Understanding the role of membrane transporters in CRC sheds light on potential diagnostic markers, therapeutic targets, and the underlying mechanisms contributing to tumorigenesis [36,37]. Membrane transporters play crucial roles in maintaining cellular homeostasis by regulating the movement of ions, nutrients, metabolites, and signaling molecules across cell membranes [38,39]. The sodium pump (Na+/K + -ATPase) presents a promising avenue for the advancement of anticancer pharmaceuticals due to its role as a signaling mediator, involvement in cell adhesion, and its association with aberrant expression and activity, which are linked to the onset and advancement of various cancer types. Focusing on Na+/K + -ATPase α subunits has the potential to usher in a novel era in anticancer treatment, bridging the gap between the aspiration of personalized medicine and its actualization [40].

A key event mediating tumor cell pH alterations is an aberrant activation of ion channels and proton pumps such as (H+)-vacuolar-ATPase (V-ATPase) [41]. Efforts aimed at identifying strategies for the development of selective inhibitors targeting V-ATPases, which play a crucial role in cancer progression, have persisted for over two decades [42].

In the context of CRC, alterations in transporter expression, function, or regulation can impact various cellular processes, including proliferation, apoptosis, migration, and drug resistance, contributing to cancer progression [9,43].

The research delved into the involvement of two specific membrane transporters, ANO7 and SLC38A4, in CRC. By analyzing vast datasets, such as TCGA, and employing various advanced methodologies like co-expression network analysis and survival correlation studies, the study aimed to understand how these transporters contribute to CRC. Key findings revealed distinct expression patterns of ANO7 and SLC38A4 in CRC tissues compared to normal tissues, indicating their potential significance in the development and progression of CRC.

ANO7 (Anoctamin 7) is a protein belonging to the TMEM16 membrane protein family, responsible for various physiological functions such as ion transport, scrambling of phospholipids, and regulation of ion channels [44]. It is expressed in various cell types, including epithelium and neurons [44]. While the role of ANO7 as an ion channel has been well-established, evolutionary analyses and related studies suggest that ANO7 may act as a multifunctional protein [45]. Studies propose its potential function as a scramblase as well [45]. Initial immunohistochemical studies indicate that ANO7 is a cell adhesion protein highly expressed at the apical pole and on lateral surfaces of epithelial cells in prostate glands; however, its precise function remains unknown.

Lee et al., in 2015 found that certain genes, such as ARL6IP5, RAET1E, and ANO7, may play crucial roles in breast cancer development and prognosis. These genes could potentially serve as predictive markers in breast cancer [46]. Studies by Kaikkonen et al., in 2018 revealed that mutations in the Anoctamin 7 (ANO7) gene could serve as an effective biomarker for early detection of advanced prostate cancer [20]. Hosseinzadeh et al., in 2020 assessed the gene expression of ANO7 in prostate cancer patients and examined its correlation with age, tumor stage, and family history of prostate cancer. The results suggest that this gene might act as a marker for the diagnosis and evaluation of prostate cancer [47]. Marks et al., in 2021 recently discovered that ANO7 is highly expressed in normal prostate glandular cells but is less abundant in cancerous cells [21]. In a recent study by Ruifang Sun et al., genes associated with CRC including ANO7 were found to be significantly upregulated in carcinoma tissues compared to their counterparts in normal and adenoma tissues, Of course, no laboratory validation was done in this study, while this issue was discussed in our study [48].

SLC38A4 is a protein belonging to the sodium-coupled neutral amino acid transporter family. This protein is expressed in various cell types, including alpha cells of the gastric mucosa, neurons, and the liver [23,[49], [50], [51]]. In 2015, Taman et al. found associations between the SLC38A4 gene and collagen degradation, collagen biosynthesis, integrin-mediated cell surface interactions, extracellular matrix organization, extracellular matrix degradation, validated targets of C-MYC transcriptional activation, collagen formation, and leukocyte migration through endothelial cells [52].

SLC38A4 has been identified in liver cancer as a tumor-suppressor gene that inhibits cell proliferation, promotes cellular apoptosis, and its molecular event of silencing serves as an oncofetal event. DNA hypermethylation leading to reduced regulation of SLC38A4 aids in liver cancer. Reduced SLC38A4 expression was accompanied by poor prognosis in liver cancer patients. Another study revealed the expression of SLC38A1, an associated amino acid transporter, in CRC [25,53,54].

Christian Pertl et al. (2005) observed that most tested genes in adenomas, except for SLC38A4, were naturally expressed in the proliferative compartment of normal mucosal cells. These results align with studies conducted on CRC cell lines [55]. Kim et al., in 2019 demonstrated an increase in SLC38A4 transporter expression in alpha cells following glucagon receptor antibody injection. The upregulation of SLC38A4 and increased alpha cell proliferation is mechanistically linked to the rapamycin-sensitive pathway [51]. Hao Kuo et al. (2022) highlighted that immunocomotaxis genes exhibit the highest differential expression in cancerous tissues and mediate interactions with immune cells. This study identified that the CAPZA1 gene regulates actin polymerization, controlling cellular motility by binding to actin filament ends. CAPZA1 is overregulated in hepatocellular carcinoma and gastric cancer. Additionally, the HMGCS2 gene is downregulated in CRC, serving as a critical downstream target for SLC38A4 to regulate tumor metabolism [56]. One of the strengths of this study is that the data obtained were derived both from biological systems and rigorously validated through laboratory experiments. Furthermore, this study could be replicated in other communities with a wider range of samples, which were restricted in this study.

## 5 Conclusion

Overall, this comprehensive study shed light on the crucial role of ANO7 and SLC38A4 in CRC, proposing their potential as diagnostic markers and therapeutic targets. The interdisciplinary approach used advanced computational analyses and experimental validation, providing valuable insights into CRC pathogenesis and paving the way for more precise approaches in cancer research and treatment.

## Funding

This work was supported by grants from Postdoctoral Research Initiation Fund from 10.13039/501100014895Southwest Medical University (Grant No. 02/00170043), Science and Technology Project of Luzhou City (Grant No. 2022-SYF-63), and Sichuan Provincial Key Laboratory for Development and Utilization of Characteristic Horticultural Biological Resources, 10.13039/501100004498Chengdu Normal University (Grant No. 2023TSYY-05).

## Data availability statement

The public data set of this study (TCGA_COAD) can be found in the TCGA database (https://portal.gdc.cancer.gov/).

## Patient consent for publication

The research protocol was assigned the Ethics Code IR.IAU.PS.REC.1402.138.

## CRediT authorship contribution statement

**Elaheh Mohandesi Khosroshahi:** Software, Methodology, Formal analysis. **Mazaher Maghsoudloo:** Writing – review & editing, Writing – original draft, Methodology, Formal analysis. **Hossein Fahimi:** Resources, Methodology, Formal analysis. **Khatere Mokhtari:** Writing – original draft, Methodology, Formal analysis. **Maliheh Entezari:** Writing – review & editing, Writing – original draft, Supervision, Conceptualization. **Maryam Peymani:** Writing – review & editing, Writing – original draft, Methodology, Conceptualization. **Mehrdad Hashemi:** Writing – review & editing, Writing – original draft, Methodology, Data curation, Conceptualization. **Runlan Wan:** Writing – review & editing, Writing – original draft, Project administration, Conceptualization.

## Declaration of competing interest

The authors declare the following financial interests/personal relationships which may be considered as potential competing interests: Runlan Wan reports financial support was provided by 10.13039/501100014895Southwest Medical University. This work was supported by grants from Postdoctoral Research Initiation Fund from 10.13039/501100014895Southwest Medical University (Grant No. 02/00170043), Science and Technology Project of Luzhou City (Grant No. 2022-SYF-63), and Sichuan Provincial Key Laboratory for Development and Utilization of Characteristic Horticultural Biological Resources, 10.13039/501100004498Chengdu Normal University (Grant No. 2023TSYY-05) If there are other authors, they declare that they have no known competing financial interests or personal relationships that could have appeared to influence the work reported in this paper.
