# Supplementary material for: Determining expression changes of ANO7 and SLC38A4 membrane transporters in colorectal cancer
Source: Heliyon. 2024 Jul 11;10(14):e34464. doi: 10.1016/j.heliyon.2024.e34464 (PMC11305260; doi:10.1016/j.heliyon.2024.e34464)
Supplement: Multimedia component 1 [file mmc1.docx]

**Table S1.** Clinical data information

| **Label** | **Age** | **Gender** | **Stage** | **TNM.T** | **TNM.N** |
| --- | --- | --- | --- | --- | --- |
| T1 | 52 | Male | II | T2 | N0 |
| T2 | 48 | Female | I | T1 | N0 |
| T3 | 62 | Male | II | T3 | N1 |
| T4 | 61 | Female | III | T3 | N1 |
| T5 | 58 | Female | IV | T4 | N2 |
| T6 | 65 | Female | I | T2 | N0 |
| T7 | 43 | Male | IV | T3 | N2 |
| T8 | 39 | Male | III | T3 | N2 |
| T9 | 51 | Male | II | T2 | N1 |
| T10 | 57 | Female | II | T2 | N0 |
| T11 | 67 | Female | II | T3 | N1 |
| T12 | 65 | Male | III | T4 | N2 |
| T13 | 45 | Female | III | T4 | N2 |
| T14 | 54 | Female | IV | T4 | N2 |
| T15 | 47 | Male | II | T2 | N1 |
| T16 | 57 | Female | II | T3 | N1 |
| T17 | 55 | Male | II | T3 | N1 |
| T18 | 58 | Female | III | T3 | N2 |
| T19 | 61 | Female | IV | T4 | N2 |
| T20 | 54 | Male | I | T1 | N0 |
| T21 | 72 | Female | III | T4 | N2 |
| T22 | 61 | Female | IV | T4 | N2 |
| T23 | 57 | Male | II | T2 | N1 |
| T24 | 43 | Male | III | T3 | N2 |
| T25 | 47 | Male | III | T4 | N2 |
