# Supplementary material for: Determining expression changes of ANO7 and SLC38A4 membrane transporters in colorectal cancer
Source: Heliyon. 2024 Jul 11;10(14):e34464. doi: 10.1016/j.heliyon.2024.e34464 (PMC11305260; doi:10.1016/j.heliyon.2024.e34464)
Supplement: Multimedia component 2 [file mmc2.docx]

**Table S2.** Sequence of specific primers used in this research

| Size Band (bp) | TM(°C) | GC% | Primer sequence (5’-3’) | Gene |
| --- | --- | --- | --- | --- |
| 215 | 54.4 | 52.4 | GTAACATCTGGTGGCTCTTCC | ANO7-F |
|  | 54.4 | 40 | TGTAGAACTCCAAGGACATCAATAC | ANO7-R |
| 134 | 53.0 | 45.5 | ACTGTTATTTCCCAAACGACCC | SLC38A4-F |
|  | 51.8 | 50 | AAGAAGCCCCTATGAATCCG | SLC38A4-R |
| 101 | 53.0 | 45.5 | ACAACTTTGGTATCGTGGAAGG | GAPDH-F |
|  | 53.2 | 57.9 | GCCATCACGCCACAGTTTC | GAPDH-R |
